# Almost Periodic Dynamics for Memristor-Based Shunting Inhibitory Cellular Neural Networks with Leakage Delays

**DOI:** 10.1155/2016/3587271

**Published:** 2016-10-19

**Authors:** Lin Lu, Chaoling Li

**Affiliations:** Research Center of Modern Enterprise Management of Guilin University of Technology, Guilin University of Technology, Guilin 541004, China

## Abstract

We investigate a class of memristor-based shunting inhibitory cellular neural networks with leakage delays. By applying a new Lyapunov function method, we prove that the neural network which has a unique almost periodic solution is globally exponentially stable. Moreover, the theoretical findings of this paper on the almost periodic solution are applied to prove the existence and stability of periodic solution for memristor-based shunting inhibitory cellular neural networks with leakage delays and periodic coefficients. An example is given to illustrate the effectiveness of the theoretical results. The results obtained in this paper are completely new and complement the previously known studies of Wu (2011) and Chen and Cao (2002).

## 1. Introduction

It is common knowledge that shunting inhibitory cellular neural networks (SICNNs) have a wide application in many fields such as image processing, signal processing, pattern recognition, psychophysics, speech, perception, robotics, and vision [1–3]. Thus, the theoretical analysis and applied research on SICNNs have attracted worldwide attention. During the past decades, memristor which is a new circuit element has received much attention due to its wide range of applications in computer, physics, electronic engineering, and so on [4, 5]. In particular, memristor has memory function and nanometer dimensions. The former can help us to deal with nanocomputing and the latter can provide a very high density and is less power hungry. The memristor can exhibit features as what the neurons in the human brain possess [4].

In practical implementation, the time delays often occur in neural networks due to the finite switching speed of the neuron amplifiers and the finite signal transmission velocity. Here, we would like to point out that a typical time delay called leakage (or forgetting) delay may occur in the negative feedback term of the neural networks and plays an important role in characterizing the dynamical behavior of neural networks [6–11]. For example, time delay in the stabilizing negative feedback term may destabilize a system [12]. Balasubramaniam et al. [13] argued that the existence and uniqueness of the equilibrium point have nothing to do with time delays and initial conditions. Thus, it is important to study the leakage delays' effect on the dynamical behavior of memristor-based neural networks. In recent years, there is some work on this topic. We refer the readers to [14–16].

As is known to us, periodic oscillation of neural networks plays an important role in the daily life of human beings. Periodic oscillation of neural networks has been widely applied in many biological and cognitive activities. For example, periodic oscillatory or chaotic phenomena often occur in the human brain. Thus, some authors investigate the periodic oscillatory dynamical behavior of neural networks for grasping the mechanism of the human brain. We refer the readers to [17–19]. However, in many cases, the periodic parameters of neural networks may experience certain perturbations and then they may be not periodic. Thus, it is more reasonable to characterize the reality of neural networks with almost periodic parameters. In recent years, many authors consider the almost periodic oscillation of neural networks with or without delay and numerous good results have been available. For example, Liu et al. [20] focused on the almost periodic solution of impulsive Hopfield neural networks with finite distributed delays by applying fixed point theorems, Lyapunov functional, and some inequality techniques. Li et al. [21] investigated the existence and global exponential stability of almost periodic solution for high-order BAM neural networks with delays on time scales. By using a fixed point theorem and by constructing a suitable Lyapunov functional, authors established some sufficient conditions to ensure the existence and global exponential stability of almost periodic solution for high-order bidirectional associative memory neural networks with delays on time scales. Huang [22] presented some sufficient conditions for the existence and exponential stability of almost periodic solutions for fuzzy cellular neural networks with time-varying delays. Li et al. [23] established some sufficient conditions to ensure the existence and stability of pseudo almost periodic solution for neutral type high-order Hopfield neural networks with delays in leakage terms on time scales by means of fixed point theorem and the theory of calculus on time scales. For more results on this aspect, we refer the readers to [24–34]. To the best of our knowledge, there are no results on the existence and stability of almost periodic solution of memristor-based shunting inhibitory cellular neural networks with leakage delays.

Inspired by the discussions above, in this article, we considered the following memristor-based shunting inhibitory cellular neural networks with leakage delays:

(1) x˙ijt=−aijtxijt−σijt−∑Ckl∈Nιi,jCijklt,xijtfxijt−τijtxijt+Lijt,

where *i* = 1,2,…, *n*,  *j* = 1,2,…, *m*,  *C*_*ij*_ represents the cell at the (*i*, *j*) position of the lattice, the *r*-neighborhood *N*_*r*_(*i*, *j*) of *C*_*ij*_ is

(2) Nri,j=Cijkl:max⁡k−l,l−j≤ι,1≤k≤m,1≤l≤n,

*x*_*ij*_ is the activity of the cell *C*_*ij*_, *L*_*ij*_(*t*) is the external input to *C*_*ij*_, the constant *a*_*ij*_ > 0 represents the passive decay rate of the cell activity, the activation function *f*(*x*_*kl*_) is a positive continuous function representing the output or firing rate of the cell *C*_*ij*_, *σ*_*ij*_(*t*) ≥ 0 and *τ*_*ij*_(*t*) ≥ 0 denote the leakage delay and transmission delay at time *t*, *C*_*ij*_^*kl*^(*t*, *x*_*ij*_(*t*)) ≥ 0 is memristive synaptic weights (which means the connection or coupling strength of postsynaptic activity of the cell transmitted to the cell *C*_*ij*_), which is defined as follows:

(3) $$\begin{array}{c} C_{ij}^{kl}\left(\;t,\xi \;\right)=\left\{\begin{array}{cc} C_{ij}^{kl*}\left(t\right), & h_{ij}\left(\xi \right)>T_{ij},\\ C_{ij}^{kl**}\left(t\right), & h_{ij}\left(\xi \right)<T_{ij}, \end{array}\right. \end{array}$$

for *t* ∈ *R*, *a*_*ij*_(*t*, *x*) = *C*_*ij*_^*kl∗*^(*t*) or *C*_*ij*_^*kl∗∗*^(*t*) when *h*_*ij*_(*ξ*) = *T*_*ij*_, when *h*_*ij*_ : *R* ^*n*+*m*^ → *R*  (*i* = 1,2,…, *n*, *j* = 1,2,…, *m*) are threshold level functions, *T*_*ij*_ ∈ *R*  (*i* = 1,2,…, *n*, *j* = 1,2,…, *m*) are threshold level, and *C*_*ij*_^*kl∗*^ and *C*_*ij*_^*kl∗∗*^ are all continuous functions. The initial states associated with (3) are given by

(4) xijs=φijs,s∈−δ,0,  φijs∈C−δ,0,  i=1,2,…,n,  j=1,2,…,m,

where *δ* = max_*t*∈*R*_{*τ*_*ij*_(*t*), *η*_*ij*_(*t*)}.

The main purpose of this article is to investigate the existence and exponential stability of the almost periodic solutions for system (1). With the aid of new Lyapunov function techniques, we establish some new sufficient criteria which guarantee the existence, uniqueness, and exponential stability of the almost periodic solution of system (1). Also, the derived results on the almost periodic solution are applied to prove the existence and stability of periodic solution for memristor-based shunting inhibitory cellular neural networks with leakage delays and periodic coefficients. The obtained results of this article are new and complement previously known publications.

The remainder of the paper is organized as follows. In Section 2, some necessary definitions and lemmas are stated. In Section 3, a set of sufficient criteria which guarantee the global existence and boundedness of any solutions and the existence and exponential stability of an almost periodic solution of neural networks (1) are established. The global exponential periodicity and stability of system (1) are analyzed in Section 4. An example is given to show the correctness of the theoretical predictions in Section 5. A brief conclusion is drawn in Section 6.

## 2. Preliminaries 

In this section, we list several definitions and notations. Suppose *E* ∈ *R* ^*n*+*m*^; then *x* → *F*(*x*) is called a set-valued map from *E* to *R* ^*n*+*m*^, if, for each point *x* ∈ *E*, there exists a nonempty set *F*(*x*) ⊂ *R* ^*n*+*m*^. A set-valued map *F* with nonempty values is said to be upper semicontinuous at *x*_0_ ∈ *E*, if, for any open set *N* containing *F*(*x*_0_), there exists a neighborhood *M* of *x*_0_ such that *F*(*M*)⊆*N*. The map *F*(*x*) is said to have a closed (convex, compact) image if, for each *x* ∈ *E*, *F*(*x*) is closed (convex, compact). For *φ* ∈ *C*(−*δ*, 0], let ‖*φ*‖ = sup_*s*∈−*δ*, 0]_‖*φ*(*s*)‖. Given the function *V* : *R* ^*n*+*m*^ → *R*,  ∇*V* denotes the gradient of *V* and ∂*V* denotes Clarke's generalized gradient of *V*.

In (1), since *C*_*ij*_^*kl*^(*t*, *ξ*) is discontinuous, the classical definition of the solution for differential equations cannot apply here. To handle this problem, Filippov developed a solution concept for the differential equation with a discontinuous right-hand side. Based on this definition, a differential equation with a discontinuous right-hand side has the same solution set as a certain differential inclusion. In what follows, we use this definition to discuss dynamical behavior of (1). Let the set-valued maps be as follows:

(5) co¯Cijklt,ξ=Cijkl∗t,hijξ>Tij,co¯Cijkl∗t,Cijkl∗∗t,hijξ=Tij,Cijkl∗∗t,hijξ<Tij,

for *t* ∈ *R*,  *i* = 1,2,…, *n*,  *j* = 1,2,…, *m*, where $\bar{co}$ denotes the convex closure of a set. Obviously, $\bar{co}(a_{ij}(t,\xi ))$ is all closed, convex, and compact in *ξ* for each *t* ∈ *R*,  *i* = 1,2,…, *n*,  *j* = 1,2,…, *m*. We define the Filippov solution of (1) as the following.

**Definition 1 .**

A function *x*(*t*) is said to be a solution of (1) on [0, *T*) with initial condition (4), if *x*(*t*) is absolutely continuous on any compact interval of [0, *T*) and satisfies differential inclusions

(6) dxijtdt∈−aijtxijt−σijt−∑Ckl∈Nιi,jco¯Cijklt,xijtfxijt−τijt·xijt+Lijt,

or, equivalently, there exist $D_{ij}^{kl}(t)\in \bar{co}(C_{ij}^{kl}(t,x_{ij}(t)))$ satisfying

(7) dxijtdt=−aijtxijt−σijt−∑Dkl∈Nιi,jDijkltfxijt−τijtxijt+Lijt,

for a.e. *t* ∈ [0, *T*], *i* = 1,2,…, *n*, *j* = 1,2,…, *m*.

**Definition 2 .**

A continuous function *x*(*t*) : *R* → *R* ^*n*+*m*^ is said to be almost periodic on *R* if, for any *ε* > 0, it is possible to find a real number *l* = *l*(*ε*) > 0. For any interval with length *l*, there exists a number *ω* = *ω*(*ε*) in this interval, such that |*x*(*t* + *ω*) − *x*(*t*)| < *ε* for all *t* ∈ *R*.

**Definition 3 .**

A continuous function *x*(*t*) : *R* → *R* ^*n*+*m*^ is said to be asymptotically almost periodic on *R* if, for any *ε* > 0, there exist *T* > 0,  *l* = *l*(*ε*), and *ω* = *ω*(*ε*) in any interval with the length of *l*, such that |*x*(*t* + *ω*) − *x*(*t*)| < *ε* for all *t* ≥ *T*.

**Definition 4 .**

The neural networks model is said to be globally exponentially almost periodic if the state *x*(*t*, *φ*) of the neural networks model is globally exponentially convergent to an almost periodic state *x* ^*∗*^(*t*, *φ*); that is, there are constants *M* ≥ 1 and *μ* > 0 such that, for any *φ* ∈ *C*(−*δ*, 0], ‖*x*(*t*, *φ*) − *x* ^*∗*^(*t*, *φ*)‖ ≤ *M*‖*φ* − *ψ*‖*e* ^−*μt*^, *t* ≥ 0. In addition, if *x* ^*∗*^(*t*, *φ*) is a periodic solution (equilibrium), then the neural networks model is said to be globally exponentially periodic (stable).

**Definition 5 (see [35]).**

*V*(*x*) : *R* ^*n*+*m*^ → *R* is said to be regular, if, for each *x* ∈ *R* ^*n*+*m*^ and *ν* ∈ *R* ^*n*+*m*^,

 (i) there exists the usual right or left directional derivative
  (8) $$\begin{array}{c} D^{+}V\left(\;x,\nu \;\right)=\underset{h\to 0^{+}}{lim}\frac{V\left(x+h\nu \right)-V\left(x\right)}{h}; \end{array}$$
 (ii) the generalized directional derivative of *V* at *x* in the direction *ν* ∈ *R* ^*n*+*m*^ is defined as
  (9) $$\begin{array}{c} D^{++}V\left(\;x,\nu \;\right)=\underset{y\to x,h\to 0^{+}}{lim}\frac{V\left(y+h\nu \right)-V\left(y\right)}{h}; \end{array}$$

then *D* ^+^ *V*(*x*, *ν*) = *D* ^++^ *V*(*x*, *ν*).

**Definition 6 .**

For a locally Lipschitz function *V* : *R* ^*n*+*m*^ → *R*, one can define Clarke's generalized gradient of *V* at point *x*, as follows:

(10) ∂Vx=co¯limk→∞⁡∇Vxk:xk⟶x,  xk∉N,  xk∉Ω,

where *Ω* ⊂ *R* ^*n*+*m*^ is the set of points where *V* is not differentiable and *N* ⊂ *R* ^*n*+*m*^ is an arbitrary set with measure zero.

**Lemma 7 (see [36]).**

If *V*(*x*) : *R* ^*n*+*m*^ → *R* is Clarke's regular and *x*(*t*):[0, +*∞*) → *R* ^*n*+*m*^ is absolutely continuous on any compact interval of [0, +*∞*), then *x*(*t*) and *V*(*x*(*t*)):[0, +*∞*) ∈ *R* ^*n*+*m*^ are differential for a.a. *t* ∈ [0, +*∞*), and one will have $dv(t)/dt=\gamma (t{)}^{T}\dot{x}(t),\forall \gamma (t)\in \partial V\left(x\left(t\right)\right)$, where ∂*V*(*x*(*t*)) is Clarke's generalized gradient.

**Lemma 8 (see [37]).**

Let matrix *M* = (*m*_*ij*_)_*n*×*n*_ have nonpositive off-diagonal elements. Then, *M* is a nonsingular *M*-matrix if and only if one of the following conditions holds:

(1) There exist *n* positive constants *α*_1_, *α*_2_,…, *α*_*n*_ such that
  (11) $$\begin{array}{c} \alpha_{i}m_{ii}-\overset{n}{\underset{j=1,j\neq i}{\sum }}\alpha_{j}\left|\;m_{ji}\;\right|>0,\;i=1,2,\ldots ,n. \end{array}$$
(2) There exist *n* positive constants *β*_1_, *β*_2_,…, *β*_*n*_ such that
  (12) $$\begin{array}{c} \beta_{i}m_{ii}-\overset{n}{\underset{j=1,j\neq i}{\sum }}\beta_{j}\left|\;m_{ij}\;\right|>0,\;i=1,2,\ldots ,n. \end{array}$$

Denote *a* ^+^ = sup_*t*∈*R*_|*a*(*t*)|, *a* ^−^ = inf_*t*∈*R*_|*a*(*t*)|, where *a* : *R* → *R* is a bounded continuous function.

Throughout this paper, we assume that the following conditions are satisfied:

 (H1) For *i* = 1,2,…, *n*, *j* = 1,2,…, *m*, *C*_*ij*_^*kl∗*^(*t*), *C*_*ij*_^*kl∗∗*^(*t*), *L*_*ij*_(*t*), *a*_*ij*_(*t*), *σ*_*ij*_(*t*), and *τ*_*ij*_(*t*) are continuous functions and are almost periodic; that is, for any *ε* > 0, it is possible to find a real number *l* = *l*(*ε*) > 0; for any interval with length *l*, there exists a number *ω* = *ω*(*ε*) in this interval, such that |*C*_*ij*_^*kl∗*^(*t* + *ω*) − *C*_*ij*_^*kl∗*^(*t*)| < *ε*,  |*C*_*ij*_^*kl∗∗*^(*t* + *ω*) − *C*_*ij*_^*kl∗∗*^(*t*)| < *ε*,  |*L*_*ij*_(*t* + *ω*) − *L*_*ij*_(*t*)| < *ε*,  |*a*_*ij*_(*t* + *ω*) − *a*_*ij*_(*t*)| < *ε*,  |*τ*_*ij*_(*t* + *ω*) − *τ*_*ij*_(*t*)| < *ε*, and  |*σ*_*ij*_(*t* + *ω*) − *σ*_*ij*_(*t*)| < *ε* for all *t* ∈ *R*.
 (H2) For *i* = 1,2,…, *n*, *j* = 1,2,…, *m*, *σ*_*ij*_(*t*) is bounded above and below by positive constants and *σ*_*ij*_′(*t*) is a bounded continuous function and *σ*_*ij*_^+^ *a*_*ij*_^+^ < 1.
 (H3) There exists constant *L* > 0 such that |*f*(*u*) − *f*(*v*)| ≤ *L*|*u* − *v*| for *u*, *v* ∈ *R*.
 (H4) There exist positive constants *δ*_11_, *δ*_12_,…, *δ*_1*m*_, *δ*_21_, *δ*_22_,…, *δ*_*nm*_ and *ς* such that
  (13) −aijt1−2aij+σij+−aijt−1−σij′taijt−σijtδkl1−akl+σkl++ ∑Ckl∈Nιi,jCijklutLδijϱ/ς1−aij+σij+2<−ς,
 for all *t* > 0 and *i*, *j* = 1,2,…, *n*, *j* = 1,2,…, *m*, where (*C*_*ij*_^*kl*^)^*u*^(*t*) = max⁡{|*C*_*ij*_^*kl∗*^(*t*)|, |*C*_*ij*_^*kl∗∗*^|(*t*)} and *ϱ* = max_1≤*i*≤*n*,1≤*j*≤*m*_{*δ*_*ij*_((*C*_*ij*_^*kl*^)^+^)^*u*^|*f*(0)| + *L*_*ij*_^+^}.
 (H5) There exists a nonempty subset Λ_*ij*_ ⊂ *R*  (*i* = 1,2,…, *n*,  *j* = 1,2,…, *m*) satisfying the following property: if *h*_*ij*_(*x*) ≤ *T*_*ij*_ ≤ *h*_*ij*_(*y*), then there exists *λ*_*ij*_ ∈ Λ_*ij*_ such that *x*_*ij*_ ≤ *λ*_*ij*_ ≤ *y*_*ij*_ or *y*_*ij*_ ≤ *λ*_*ij*_ ≤ *x*_*ij*_.
 (H6) For *i* = 1,2,…, *n*, *j* = 1,2,…, *m*, *f*(*λ*_*ij*_) = 0 for any *λ*_*ij*_ ∈ Λ_*ij*_.

## 3. Boundedness and Almost Periodicity

In this section, we will prove the existence of bounded solution and the exponential stability of almost periodic solution for (1).

**Theorem 9 .**

Assume that assumptions (H1)–(H4) hold. Let *x*(*t*) be the solution of (1) with initial condition

(14) xijt=φijt,φijt−∫t−σijttaijsφijsds<δijϱς,t∈−δ,0,

where *i* = 1,2,…, *n*, *j* = 1,2,…, *m*, and

(15) $$\begin{array}{c} \varrho =\underset{1\leq i\leq n,1\leq j\leq m}{max}\left\{\;\delta_{ij}{\left(\;{\left(\;C_{ij}^{kl}\;\right)}^{+}\;\right)}^{u}\left|\;f\left(\;0\;\right)\;\right|+L_{ij}^{+}\;\right\}. \end{array}$$

Then,

(16) xijt−∫t−σijttaijsxijsds<δijϱς,

(17) xijt≤δijϱ/ς1−aij+σij+,

where *t* is in the interval of existence and *i* = 1,2,…, *n*, *j* = 1,2,…, *m*.

**Proof**

Let *x*(*t*) be a solution of (1) with initial condition (14). For *i* = 1,2,…, *n*, *j* = 1,2,…, *m*, there exists $D_{ij}^{kl}(t)\in \bar{co}\left(C_{ij}^{kl}\left(t,x_{ij}\left(t\right)\right)\right)$ satisfying

(18) x˙ijt=−aijtxijt−σijt−∑Dkl∈Nιi,jDijkltfxijt−τijtxijt+Lijt,

for a.a. *t* ∈ [0, *T*]. By (5) and (H3), we have

(19) DijkltmaxCijkl∗t,Cijkl∗∗t≤Cijklu,fxijtfxijt−f0+f0≤Lxijt+f0.

For *t* in the interval of existence and *i* = 1,2,…, *n*, *j* = 1,2,…, *m*, denote

(20) $$\begin{array}{c} u_{ij}\left(\;t\;\right)=x_{ij}\left(\;t\;\right)-\overset{t}{\underset{t-\sigma_{ij}\left(t\right)}{\int }}a_{ij}\left(\;s\;\right)x_{ij}\left(\;s\;\right)ds. \end{array}$$

Suppose (16) holds; then, for a given *t* ^*∗*^ > 0 in the interval of existence and *i* = 1,2,…, *n*, *j* = 1,2,…, *m*, we get

(21) xijt∗uijt∗+∫t∗−σijt∗t∗aijsxijsds≤δijϱς+aij+σij+sups∈−δ,t∗⁡xijs

for any *t* ^*∗*^ ∈ (−*δ*, *t*]. Then,

(22) $$\begin{array}{c} \underset{s\in \left(-\delta ,t^{*}\right]}{sup}\left|\;x_{ij}\left(\;s\;\right)\;\right|<\delta_{ij}\frac{\varrho }{\varsigma }+a_{ij}^{+}\sigma_{ij}^{+}\underset{s\in \left[-\delta ,t^{*}\right]}{sup}\left|\;x_{ij}\left(\;s\;\right)\;\right| \end{array}$$

for any *t* ^*∗*^ ∈ (−*δ*, *t*]. It follows from (22) that

(23) $$\begin{array}{c} \left|\;x_{ij}\left(\;t\;\right)\;\right|\leq \frac{\delta_{ij}\left(\varrho /\varsigma \right)}{1-a_{ij}^{+}\sigma_{ij}^{+}}. \end{array}$$

Therefore, (17) holds. Thus, it suffices to prove (16). Assume that (16) does not hold. Then, there exist *k* ∈ {1,2,…, *n*},  *l* ∈ {1,2,…, *m*}, and *t*_0_ > 0 such that |*u*_*kl*_(*t*_0_)| = *δ*_*kl*_(*ϱ*/*ς*) and (16) holds for all *t* ∈ [−*δ*, *t*_0_] and *i* = 1,2,…, *n*,  *j* = 1,2,…, *m* and hence *D* ^−^|*u*_*kl*_(*t*_0_)| ≥ 0. From system (1), we have

(24) dukltdt=xkl′t−akltxklt−1−σkl′t·aklt−σkltxklt−σklt=−akltxklt−1−σkl′taklt−σkltxklt−σklt−akltxklt−σklt−∑Dkl∈Nri,jDijklt·fxklt−τkltxklt+Lklt=−aklt·xklt−aklt−1−σkl′taklt−σklt·xklt−σklt−∑Dkl∈Nri,jDijklt·fxklt−τkltxklt+Lklt=−aklt·uklt−aklt∫t−σklttaklsxklsds−aklt−1−ηkl′taklt−σklt·xklt−σklt−∑Dkl∈Nri,jDijklt·fxklt−τkltxklt+Lklt.

It follows from (H3) that

(25) D−uklt0≤−aklt0uklt0+aklt0·∫t−σklt0t0aklsxklsds+aklt0−1−σkl′t0aklt0−σklt0·xklt0−σklt0+∑Dkl∈Nri,jDijklt0·fxklt0−τklt0xklt0+Lklt0≤−aklt0uklt0+aklt0∫t−σklt0t0akls·xklsds+aklt0−1−σkl′t0aklt0−σklt0xklt0−σklt0+∑Dkl∈Nri,jDijklt0·Lxklt0−τklt0+f0xklt0+Lklt0≤−aklt0δklϱς+aklt0akl+ηkl+·δklϱ/ς1−akl+σkl++aklt0−1−σkl′t0aklt0−σklt0δklϱ/ς1−akl+σkl++∑Dkl∈Nri,jDijklt0·Lδklϱ/ς1−akl+σkl++f0δklϱ/ς1−akl+σkl++Lkl+≤−aklt01−2akl+σkl+−aklt0−1−σkl′t0aklt0−σklt0·δkl1−akl+σkl++∑Dkl∈Nri,jDijklut0·Lδklϱ/ς1−akl+σkl+2ϱς+ϱ<0,

which is a contradiction and shows that (16) holds. The proof of Theorem 9 is complete.

**Lemma 10 .**

Assume that (H3), (H5), and (H6) hold; then, for any *x* = (*x*_11_, *x*_12_,…, *x*_*nm*_)^*T*^,  *y* = (*y*_11_, *y*_12_,…, *y*_*nm*_)^*T*^ ∈ *R* ^*nm*^, one has

(26) co¯Cijklt,xijtfxij−co¯Cijklt,yijtfyij≤CijklutLxij−yij,

where *i* = 1,2,…, *n*,  *j* = 1,2,…, *m*.

**Proof**

For any given *i* ∈ {1,2,…, *n*},  *j* ∈ {1,2,…, *m*}, and *x*_*ij*_, *y*_*ij*_ ∈ *R* ^*n*+*m*^, we consider three cases.

If *h*_*ij*_(*x*), *h*_*ij*_(*y*) < *T*_*ij*_, then

(27) co¯Cijklt,xijtfxij−co¯Cijklt,yijtfyij=Cijkl∗tfxij−Cijkl∗tfyij≤CijklutLxij−yij.

If *h*_*ij*_(*x*), *h*_*ij*_(*y*) > *T*_*ij*_, then

(28) co¯Cijklt,xijtfxij−co¯Cijklt,yijtfyij=Cijkl∗∗tfxij−Cijkl∗∗tfyij≤CijklutLxij−yij.

If *h*_*ij*_(*x*) ≤ *T*_*ij*_ ≤ *h*_*ij*_(*y*) or *h*_*ij*_(*y*) ≤ *T*_*ij*_ ≤ *h*_*ij*_(*x*), then it follows from (H4) that there exists *λ*_*j*_ ∈ Λ_*j*_ such that *x*_*ij*_ ≤ *λ*_*ij*_ ≤ *y*_*ij*_ or *y*_*ij*_ ≤ *λ*_*ij*_ ≤ *x*_*ij*_. Let *x*_*ij*_ ≤ *λ*_*ij*_ ≤ *y*_*ij*_. In this case, from (H5), we get

(29) co¯Cijklt,xijtfxij−co¯Cijklt,yijtfyij=Cijkl∗∗tfxij−Cijkl∗tfyij≤Cijkl∗∗tfxij−fλj+Cijkl∗tfλj−fyij≤CijklutLxij−yij.

Based on all the cases above, we can conclude that (26) holds. The proof of Lemma 10 is complete.

Now, we state our main result.

**Theorem 11 .**

If (H1)–(H6) hold, then there exists a unique almost periodic solution *x* ^*∗*^(*t*, *ψ*) for system (1) which is globally exponentially stable; that is, for any other solution *x*(*t*, *ψ*) of system (1), there exist constants *M*, *μ* > 0 such that ‖*x*(*t*, *φ*) − *x* ^*∗*^(*t*, *ψ*)‖ ≤ *M*‖*φ* − *ψ*‖*e* ^−*μt*^ for all *t* > 0.

**Proof**

First, we prove that any solution of (1) is asymptotically almost periodic; that is, for any *ε* > 0, there exist *T* > 0,  *l* = *l*(*ε*), and *ω* = *ω*(*ε*) in any interval with the length of *l*, such that |*x*(*t* + *ω*) − *x*(*t*)| ≤ *ε* for all *t* ≥ *T*.

For any *ε* > 0, let *ω* = *ω*(*ε*) and *y*_*ij*_(*t*) = *x*_*ij*_(*t* + *ω*) − *x*_*ij*_(*t*),  *i* = 1,2,…, *n*,  *j* = 1,2,…, *m*, and then we get

(30) dyijtdt∈−aijtxijt+ω−σijt+ω+Lijt+ω−∑Ckl∈Nιi,jco¯Cijklt+ω,xijt+ω·fxijt+ω−τijt+ωxijt+ω+aijt·xijt−σijt−Lijt+∑Ckl∈Nιi,jco¯Cijklt,xijtfxijt−τijt·xijt=−aijtyijt+Aijt,xt,yt+Θijt,ω,

where *A*_*ij*_(*t*, *x*(*t*), *y*(*t*)) and Θ_*ij*_(*t*, *ω*) are defined as follows:

(31) Aijt,xt,yt=co¯Cijklt,u+vfu+v−co¯Cijklt,ufu,

for all *u*, *v* ∈ *R* ^*n*+*m*^ and

(32) Θijt,ω=−aijt+ω−aijt+ω−σijt+Lijt+ω−Lijt+∑Ckl∈Nιi,jco¯Cijklt,xijt·fxijt−τijtxijt−∑Ckl∈Nιi,jco¯Cijklt+ω,xijt+ω·fxijt+ω−τijt+ωxijt+ω.

In view of (H1) and the boundedness of *x*(*t*), we can conclude that, for any *ε* > 0, there exist *l* = *l*(*ε*) > 0, and *ω* = *ω*(*ε*) in any interval with the length of *l*, such that, for any $\Theta^{*}(t,\omega )\in \bar{co}(\Theta (t,\omega ))$, |Θ^*∗*^(*t*, *ω*)| < *Nε*/2 for all *t* ≥ 0, where *N* > 0 is a constant. Let

(33) dyijtdt=−aijtyijt+Aij∗t,xt,yt+Θij∗t,ω,

where $A_{ij}^{*}(t,x(t),y(t))\in \bar{co}(A_{ij}(t,x(t),y(t)))$ and $\Theta^{*}(t,\omega )\in \bar{co}(\Theta (t,\omega ))\;\;\left(i=1,2,\ldots ,n,\;\;j=1,2,\ldots ,m\right).$

In view of (H3), we can choose *r* > 0 and *ς* > 0 such that

(34) −aklt−r1−2akl+σkl+−aklt−1−σkl′taklt−σkltδkl1−akl+σkl++∑Ckl∈Nιi,jCijkluterτijtLδklϱ/ς1−akl+σkl+2<−ς.

Let *T*_0_ ≥ max{0, *ω*}. For *i* = 1,2,…, *n*, *j* = 1,2,…, *m* and *t* ∈ *R*, denote

(35) $$\begin{array}{c} U_{ij}\left(\;t\;\right)=e^{rt}y_{ij}\left(\;t\;\right)-\overset{t}{\underset{t-\sigma_{ij}\left(t\right)}{\int }}a_{ij}\left(\;s\;\right)e^{rs}y_{ij}\left(\;s\;\right)ds. \end{array}$$

Then, for *t* ≥ *T*_0_ and *i* = 1,2,…, *n*,  *j* = 1,2,…, *m*, we have

(36) dUijtdt=rertyijt+ertyij′t−aijtertyijt+1−σij′taijt−σijtert−σijtyijt−σijt=−aijt−rertyijt+ert−aijt−1−σij′taijt−σijte−rσijt×yijt−σijt+Aij∗t,xt,yt+Θij∗t,ω=−aijt−rUijt−aijt−r∫t−σijttaijs·ersyijsds+ert−aijt−1−σij′taijt−σijte−rσijt×yijt−σijt+Aij∗t,xt,yt+Θij∗t,ω.

Now, we define a candidate Lyapunov function as follows:

(37) W1t=maxUijtδij,  i=1,2,…,n,  j=1,2,…,m,W1∗t=sups≤t W1s.

Obviously, *W*_1_^*∗*^(*t*) is nondecreasing. It follows that

(38) erρyijρδij−1≤erρyijρ−∫ρ−σijρρaijsersyijsdsδij−1+∫ρ−σijρρaijsersyijsdsδij−1≤W1∗t+aij+σij+sups≤t ersyijsδij−1

for all *ρ* ≤ *t*, where *i* = 1,2,…, *n*, *j* = 1,2,…, *m*. By (H3), we have

(39) $$\begin{array}{c} e^{r\rho }\left|\;y_{ij}\left(\;\rho \;\right)\;\right|\delta_{ij}^{-1}\leq \frac{W_{1}^{*}\left(t\right)}{1-a_{ij}^{+}\sigma_{ij}^{+}} \end{array}$$

for all *t* ≥ 0, *ρ* ≤ *t*, where *i* = 1,2,…, *n*, *j* = 1,2,…, *m*. For any given *t* ≥ 0, there exist *k* ∈ {1,2,…, *n*},  *l* ∈ {1,2,…, *m*} such that

(40) $$\begin{array}{c} W_{1}\left(\;t\;\right)=\frac{\left|U_{kl}\left(t\right)\right|}{\delta_{kl}}. \end{array}$$

Calculating the derivative *dW*_1_(*t*)/*dt* along the positive half trajectory of (1) yields

(41) dW1tdt≤−aklt−rW1t+1δkl−aklt−r·∫t−σklttaklsersyklsds+ert−aklt−1−σkl′taklt−σklte−rσklt×yklt−σklt+Akl∗t,xt,yt+Θkl∗t,ω≤−aklt−rW1t+aklt−rσkl+akl+·W1∗t1−σkl+akl++aklterσklt−1−σkl′taklt−σkltW1∗t1−σkl+akl++1δkl∑Ckl∈Nιi,jCijklut·LerτkltW1∗t1−akl+σkl+2+ertΘkl∗t,ω≤ertδklΘkl∗t,ω−aklt−r1−2σkl+akl+−aklerσklt−1−σkl′taklt−σklt·δkl1−σkl+akl+−∑Ckl∈Nιi,jCijklut·Lerτkltδkl1−akl+σkl+2W1tδkl≤ertδklΘkl∗t,ω≤Nε2δminert

when *W*_1_^*∗*^(*t*) ≤ *W*_1_(*t*). Thus,

(42) $$\begin{array}{c} \frac{dW_{1}^{*}\left(t\right)}{dt}\leq \frac{N\varepsilon }{2\delta_{min}}e^{rt},\;\forall t\in \left[0,T\right). \end{array}$$

Then, for all *i* = 1,2,…, *n*, *j* = 1,2,…, *m*, we have

(43) yijtδijW1∗tert1−σij+aij+≤δijW1∗0ertmin1≤i≤n,1≤j≤m1−σij+aij++δijNε2δmin.

Thus, there exists a constant *T* > 0 such that, for any *t* > *T*,

(44) $$\begin{array}{c} \left\|\;y\left(\;t\;\right)\;\right\|\leq \frac{N\varepsilon }{r\delta_{min}}\overset{n}{\underset{i=1}{\sum }}\;\overset{m}{\underset{j=1}{\sum }}\delta_{ij}. \end{array}$$

Taking 0 < *N* < *rδ*_min_/∑_*i*=1_^*n*^ *δ*_*i*_, we have ‖*y*(*t*)‖ < *ε* for any *t* > *T*. Namely, for any *T* > 0, there exist *T* > 0, *l* = *l*(*ε*) > 0, and *ω* = *ω*(*ε*) in any interval with the length of *l*, such that ‖*x*(*t* + *ω*) − *x*(*t*)‖ < *ε* for all *t* ≥ *T*. Therefore, any solution *x*(*t*) of (1) with initial condition (14) is asymptotically almost periodic.

Next, we prove that there exists at least one almost periodic solution of (1).

Let *x*(*t*) be any solution of (1) with initial conditions (4) and (14). It is easy to see that, for any sequence {*t*_*k*_}_*k*∈*N*_ satisfying lim_*t*→*t*_*k*__ = +*∞*, the sequence {*x*(*t* + *t*_*k*_)}_*k*∈*N*_ is equicontinuous and uniformly bounded. In view of Arzela-Ascoli theorem and diagonal selection principle, we can select a subsequence of {*t*_*k*_} (still denoted by {*t*_*k*_}), such that *x*(*t* + *t*_*k*_) uniformly converges to a continuous function *x* ^*∗*^(*t*) on any compact set of *R*. We next prove that *x* ^*∗*^(*t*) is a solution of (1).

Let *z*_*ij*_(*t*, *t*_*k*_) = *x*_*ij*_(*t* + *t*_*k*_) − *x*_*ij*_^*∗*^(*t*) and

(45) Θ¯ijt,tk=Θijt,tk−aijtzijt,tk+co¯Aijt,xij∗t,zijt,tk;

then

(46) co¯Θ¯ijt,tk≤co¯Θijt,tk+aij+zijt,tk+∑Ckl∈Nιi,jCijkl+uzijt,tk.

In view of the boundedness of *x*(*t*), we can select the sequence {*t*_*k*_} satisfying |Θ_*ij*_^*∗*^(*t*, *t*_*k*_)| ≤ 1/*k* for any $\Theta_{ij}^{*}(t,t_{k})\in \bar{co}(\Theta_{ij})$ and all *t* ≥ 0. From this and (48), we have ${lim}_{k\to +\infty }\bar{co}({\bar{\Theta }}_{ij}(t,t_{k}))=0$ for all *t* ≥ 0 and *i* = 1,2,…, *n*, *j* = 1,2,…, *m*. Applying Lebesgue's dominated convergence theorem, we have

(47) x∗t+f−x∗t=limk→∞xtk+f−x∗tk=limk→∞∫tt+fx˙s+tkds∈limk→∞∫tt+f−aijs+tkxijs+tk−σijs+tk−∑Ckl∈Nιi,jco¯Cijkls+tk,xijs+tk·fxijs+tk−τijs+tk×xijs+tk+Lijs+tkds∈∫tt+flimk→∞co¯Θij∗t,tk−aijsxij∗t−σijs−∑Ckl∈Nιi,jco¯Cijkls,xij∗s·fxij∗sτijs+Lijsds=∫tt+f−aijsxij∗t−σijs−∑Ckl∈Nιi,jco¯Cijkls,xij∗sfxij∗sτijs+Lijsds

for all *t* ∈ *R* and *f* ∈ *R*. Thus, *x* ^*∗*^(*t*) is a solution of (1).

Here, we will prove that *x* ^*∗*^(*t*) is the almost periodic solution of (1). By the proof of the above step, for any *ε* > 0, there exist *T* > 0,  *l* = *l*(*ε*), and *ω* = *ω*(*ε*) in any interval with the length of *l*, such that |*x*(*t* + *ω*) − *x*(*t*)| ≤ *ε* for all *t* ≥ *T*. Hence, there exists sufficiently large constant *K* > 0 such that |*x*(*t* + *t*_*k*_ + *ω*) − *x*(*t* + *t*_*k*_)| ≤ *ε* for all *t* ∈ *R* and *k* > *K*. Let *k* → +*∞*, and then it is easy to obtain that |*x* ^*∗*^(*t* + *ω*) − *x* ^*∗*^(*t*)| ≤ *ε* for all *t* ∈ *R*; that is, *x* ^*∗*^(*t*) is the almost periodic solution of (1). Finally, we mainly prove that the almost periodic solution of (1) is unique and globally exponentially stable.

Let *x*(*t*) be any solution of (1) with initial conditions (4) and (14), and let *x* ^*∗*^(*t*) be an almost periodic solution of (1); that is,

(48) dxijtdt∈−aijtxijt−σijt−∑Ckl∈Nιi,jco¯Cijklt,xijtfxijt−τijt·xijt+Lijt,

(49) dxij∗tdt∈−aijtxij∗t−σijt−∑Ckl∈Nιi,jco¯Cijklt,xij∗tfxij∗t−τijt·xij∗t+Lijt.

Let *w*_*ij*_(*t*) = *x*_*ij*_(*t*) − *x*_*ij*_^*∗*^(*t*), and then

(50) duijtdt∈−aijtwij∗t−σijt−co¯Aijt,xij∗t−τijt,wijt−τijt,

where *A*_*ij*_(*t*, *x*_*j*_^*∗*^(*t*), *u*_*j*_(*t*)) is defined by (31). Let

(51) duijtdt=−aijtwij∗t−σijt−co¯Aijt,xij∗t−τijt,wijt−τijt,

where $A_{ij}\left(t,x_{ij}\left(t-\tau_{ij}\left(t\right)\right),u_{i}j\left(t-\tau_{ij}\left(t\right)\right)\right)\in \bar{co}\left(A_{ij}\left(t,x_{ij}\left(t-\tau_{ij}\left(t\right)\right),u_{ij}\left(t-\tau_{ij}\left(t\right)\right)\right)\right)$ *i*, *j* = 1,2,…, *n*, *j* = 1,2,…, *m*.

For *i* = 1,2,…, *n*, *j* = 1,2,…, *m* and *t* ∈ *R*, denote

(52) $$\begin{array}{c} W_{ij}\left(\;t\;\right)=e^{rt}w_{ij}\left(\;t\;\right)-\overset{t}{\underset{t-\sigma_{ij}\left(t\right)}{\int }}a_{ij}\left(\;s\;\right)e^{rs}w_{ij}\left(\;s\;\right)ds. \end{array}$$

Then, for *t* ≥ *T*_0_ and *i* = 1,2,…, *n*, *j* = 1,2,…, *m*, we have

(53) dWijtdt=−aijt−rWijt−aijt−r·∫t−σijttaijserswijsds+ert−aijt−1−σij′taijt−σijte−rσijt×wijt−σijt+ertAijt,x∗t,wt.

We define another candidate Lyapunov function as follows:

(54) V2t=max⁡Wijtδij,  i=1,2,…,n,  j=1,2,…,m,V2∗t=sups≤t V2s.

Obviously, *V*_2_^*∗*^(*t*) is nondecreasing. It follows that

(55) erρwijρδij−1≤erρwijρ−∫ρ−σijρρaijserswijsdsδij−1+∫ρ−σijρρaijserswijsdsδij−1≤W1∗t+aij+σij+sups≤t erswijsδij−1

for all *ρ* ≤ *t*, where *i* = 1,2,…, *n*, *j* = 1,2,…, *m*. By (H3), we have

(56) $$\begin{array}{c} e^{r\rho }\left|\;w_{ij}\left(\;\rho \;\right)\;\right|\delta_{ij}^{-1}\leq \frac{W_{2}^{*}\left(t\right)}{1-a_{ij}^{+}\sigma_{ij}^{+}}. \end{array}$$

For any given *t* ≥ 0, there exist *k* ∈ {1,2,…, *n*} and *l* ∈ {1,2,…, *m*} such that

(57) $$\begin{array}{c} V_{2}\left(\;t\;\right)=\frac{\left|W_{kl}\left(t\right)\right|}{\delta_{kl}}. \end{array}$$

Calculating the derivative *dV*_2_(*t*)/*dt* along the positive half trajectory of (1) yields

(58) dV2tdt≤−aklt−rV2t+1δkl∫t−σklttakls·erswklsds+ert−aklt−1−σkl′taklt−σklte−rσklt×wklt−σklt+Aklt,x∗t,wt≤−aklt−r·V2t+aklt−rσkl+akl+V2∗t1−σkl+akl++aklt·erσklt−1−σkl′taklt−σkltV2∗t1−σkl+akl++1δkl∑Ckl∈Nιi,jCijklutLerτkltV2∗t1−akl+σkl+2≤−aklt−r1−2σkl+akl+−aklerσklt−1−σkl′taklt−σkltδkl1−σkl+akl+−∑Ckl∈Nιi,jCijklutLerτkltδkl1−akl+σkl+2·V2tδkl≤ςV2tδkl<0

when *W*_2_^*∗*^(*t*) ≤ *W*_2_(*t*). Thus,

(59) $$\begin{array}{c} \frac{dV_{2}^{*}\left(t\right)}{dt}\leq 0,\;\forall t\in R. \end{array}$$

Then, for all *i* = 1,2,…, *n*, *j* = 1,2,…, *m* and for any *t* > 0, we have

(60) $$\begin{array}{c} \left|\;w_{ij}\left(\;t\;\right)\;\right|\leq \frac{\delta_{ij}V_{2}^{*}\left(t\right)e^{-rt}}{1-\sigma_{ij}^{+}a_{ij}^{+}}\leq \frac{\delta_{ij}V_{2}^{*}\left(0\right)e^{-rt}}{1-\sigma_{ij}^{+}a_{ij}^{+}}. \end{array}$$

Thus, for any *t* > 0, we have

(61) $$\begin{array}{c} \left\|\;x\left(\;t\;\right)-x^{*}\left(\;t\;\right)\;\right\|\leq M\left\|\;\varphi -\psi \;\right\|e^{-rt}, \end{array}$$

where

(62) $$\begin{array}{c} M=\overset{n}{\underset{i=1}{\sum }}\;\overset{m}{\underset{j=1}{\sum }}\frac{\delta_{ij}}{\left(1-\sigma_{ij}^{+}a_{ij}^{+}\right)\delta_{min}} \end{array}$$

and *x*(*t*) = *x*(*t*, *φ*) and *x* ^*∗*^(*t*) = *x* ^*∗*^(*t*, *ψ*). The proof of Theorem 11 is complete.

## 4. Periodicity and Stability

In this section, we will analyze the global exponential periodicity and stability of (1). By Theorem 11, we have the following results.

**Theorem 12 .**

In addition to (H1)–(H6), if *C*_*ij*_^*kl∗*^(*t*), *C*_*ij*_^*kl∗∗*^(*t*), *L*_*ij*_(*t*), *a*_*ij*_(*t*), *τ*_*ij*_(*t*), and *σ*_*ij*_(*t*) are *ω*-periodic functions, then there exists a unique *ω*-periodic solution *x* ^*∗*^(*t*, *ψ*) for (1) which is globally exponentially stable.

Next, we consider the following special form of (1):

(63) x˙ijt=−aijxijt−σij−∑Ckl∈Nιi,jCijklt,xijtfxijt−τijxijt+Lij,

where *i* = 1,2,…, *n*, *j* = 1,2,…, *m* and *C*_*ij*_^*kl∗*^, *C*_*ij*_^*kl∗∗*^, *L*_*ij*_, *a*_*ij*_, and *τ*_*ij*_ are all constants and

(64) $$\begin{array}{c} C_{ij}^{kl}\left(\;t,\xi \;\right)=\left\{\begin{array}{cc} C_{ij}^{kl*}\left(t\right), & h_{ij}\left(\xi \right)>T_{ij},\\ C_{ij}^{kl**}\left(t\right), & h_{ij}\left(\xi \right)<T_{ij}, \end{array}\right. \end{array}$$

for *t* ∈ *R*,  *a*_*ij*_(*t*, *x*) = *C*_*ij*_^*kl∗*^(*t*) or *C*_*ij*_^*kl∗∗*^(*t*) when *h*_*ij*_(*ξ*) = *T*_*ij*_. Let *E* ^*∗*^ = (*e*_*ij*_)_*n*×*n*_, where

(65) $$\begin{array}{c} e_{ij}=\left\{\begin{array}{cc} \frac{1}{1-a_{ij}^{+}\sigma_{ij}^{+}}\left[a_{ij}\left(1-2a_{ij}\sigma_{ij}\right)-\underset{C^{kl}\in N_{\iota }\left(i,j\right)}{\sum }\left|{\left(C_{ij}^{kl}\right)}^{u}\right|L\right] & \text{for }i=j,\\ -\frac{1}{1-a_{ij}^{+}\sigma_{ij}^{+}}\underset{C^{kl}\in N_{r}\left(i,j\right)}{\sum }{\left(C_{ij}^{kl}\right)}^{u}L & \text{for }i\neq j. \end{array}\right. \end{array}$$

By Theorem 11 and Lemma 8, we have the following result.

**Theorem 13 .**

In addition to (H1)–(H6), further assume that the matrix *E* ^*∗*^ is a nonsingular *M*-matrix. Then, there exists a unique equilibrium *x* ^*∗*^ for (63) which is globally exponentially stable; namely, for any other solution *x*(*t*, *φ*) of (63), there exist constants *M*, *r* > 0 such that ‖*x*(*t*, *φ*) − *x* ^*∗*^‖ ≤ *M*‖*φ* − *ψ*‖*e* ^−*rt*^ for all *t* > 0.

**Remark 14 .**

In [1], Wu investigated the pseudo almost periodic solution of shunting inhibitory cellular neural networks with time-varying delay (without leakage delay). In [2], Chen and Cao studied the almost periodic solution of shunting inhibitory cellular neural networks with constant delay. In this paper, we study the almost periodic solutions for memristor-based shunting inhibitory cellular neural networks with leakage delays which is different from the work of [1, 2]. All the obtained results in [1, 2] cannot be applied to system (1) to obtain the exponential stability of almost periodic solutions for memristor-based shunting inhibitory cellular neural networks (1), which implies that our results obtained in this paper are completely new and complement the previous studies to some extent.

## 5. Examples 

In this section, we present an example to verify the analytical predictions obtained in the previous section. Consider the following memristor-based shunting inhibitory cellular neural networks:

(66) x˙11t=−a11tx11t−σ11t−∑Ckl∈N11,1C11klt,x11tfx11t−τ11t·x11t+L11t,x˙12t=−a12tx12t−σ12t−∑Ckl∈N11,2C12klt,x12tfx12t−τ12t·x12t+L12t,x˙21t=−a21tx21t−σ21t−∑Ckl∈N12,1C21klt,x21tfx21t−τ21t·x21t+L21t,x˙22t=−a22tx22t−σ22t−∑Ckl∈N12,2C22klt,x22tfx22t−τ22t·x22t+L22t,

where *gf*(*x*) = tanh⁡(|*x*| − 1),  *a*_11_(*t*) = 1/10,  *a*_12_(*t*) = 1/12,  *a*_21_(*t*) = 1/15,  *a*_22_(*t*) = 1/20,  $L_{11}(t)=0.2\left(\cos\sqrt{2}t+\sin\sqrt{5}t\right)$,  $L_{12}(t)=0.1\left(\cos\sqrt{3}t+\sin\sqrt{7}t\right)$,  $L_{21}(t)=0.6\left(\cos\sqrt{11}t+\sin\sqrt{3}t\right)$,  $L_{22}(t)=0.4\left(\cos\sqrt{11}t+\sin\sqrt{13}t\right),\;\;\tau_{11}(t)=\cos^{2}t/18$,  *τ*_12_(*t*) = sin^2^ *t*/15,  *τ*_21_(*t*) = sin^2^ *t*/17,  *σ*_22_(*t*) = sin^2^ *t*/15,  *σ*_11_(*t*) = sin^2^ *t*/7,  *σ*_12_(*t*) = cos^2^ *t*/9, *σ*_21_(*t*) = cos^2^ *t*/23,  *σ*_22_(*t*) = cos^2^ *t*/19, and

(67) C11klt,ξ=0.4cos⁡t,ξ>1,unchanged,ξ=1,0.3sin⁡t,ξ<1,C12klt,ξ=cos⁡5t,ξ>1,unchanged,ξ=1,sin⁡3t,ξ<1,C21klt,ξ=0.1cos⁡3t,ξ>1,unchanged,ξ=1,0.4sin⁡5t,ξ<1,C22klt,ξ=cos⁡7t,ξ>1,unchanged,ξ=1,0.2sin⁡t,ξ<1.

Then,

(68) Ł=1,max1≤i≤n,1≤j≤maij+σij+≤170<1.

Let *δ*_11_ = 0.2,  *δ*_12_ = 0.3,  *δ*_21_ = 0.32,  *δ*_22_ = 0.41, and  *ς* = 0.3. Then,

(69) −a11t1−2a11+σ11+−a11t−1−σ11′ta11t−σ11t·δ111−a11+σ11++∑C11∈N11,1C1111ut·Lδ11ϱ/ς1−a11+σ11+2=−0.3089<−0.3=−ς,−a12t1−2a12+σ12+−a12t−1−σ12′ta12t−σ12t·δ121−a12+σ12++∑C12∈N11,2C1212ut·Lδ12ϱ/ς1−a12+σ12+2=−0.4125<−0.3=−ς,−a21t1−2a21+σ21+−a21t−1−σ21′ta21t−σ21t·δ211−a21+σ21++∑C21∈N12,1C2121ut·Lδ21ϱ/ς1−a21+σ21+2=−0.3907<−0.3=−ς,−a22t1−2a22+σ22+−a22t−1−σ22′ta22t−σ22t·δ221−a22+σ22++∑C22∈N12,2C2222ut·Lδ22ϱ/ς1−a22+σ22+2=−0.5128<−0.3=−ς.

**Figure 1:**
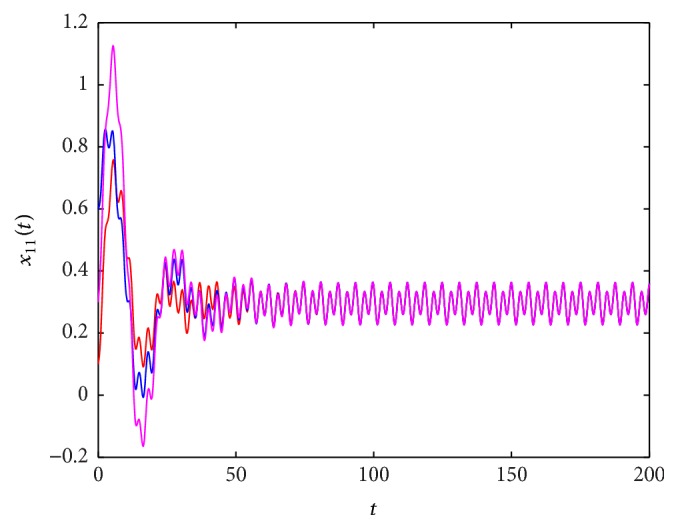
The state trajectory (*t*, *x*_11_(*t*)) of system (66) with the initial values (0.1,0.2,0.5,0.3), (0.6,0.2,0.2,0.9), and (0.3,0.8,0.9,0.7).

**Figure 2 fig2:**
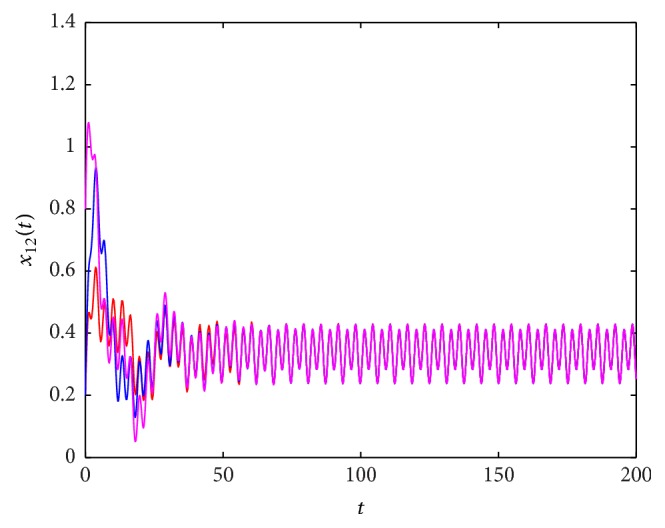
The state trajectory (*t*, *x*_12_(*t*)) of system (66) with the initial values (0.1,0.2,0.5,0.3), (0.6,0.2,0.2,0.9), and (0.3,0.8,0.9,0.7).

**Figure 3 fig3:**
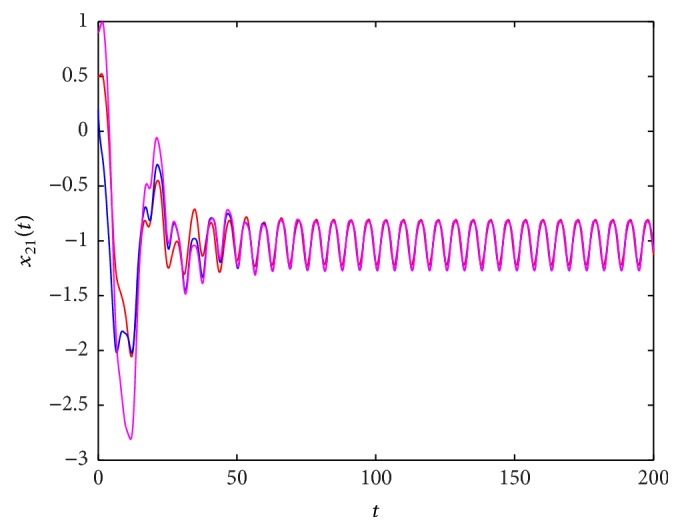
The state trajectory (*t*, *x*_21_(*t*)) of system (66) with the initial values (0.1,0.2,0.5,0.3), (0.6,0.2,0.2,0.9), and (0.3,0.8,0.9,0.7).

**Figure 4:**
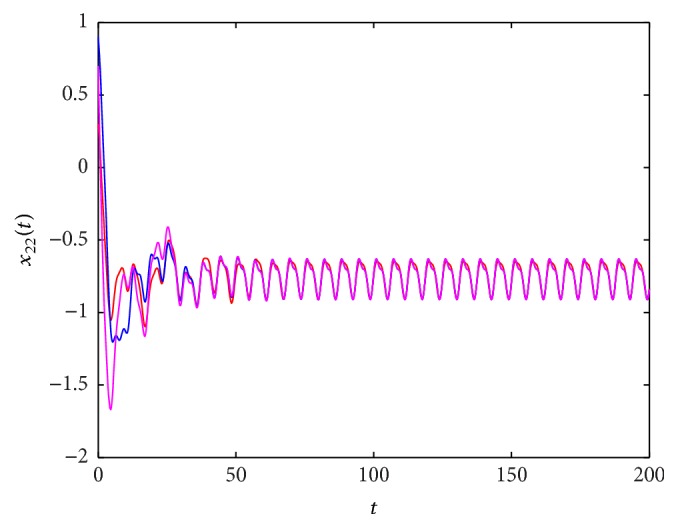
The state trajectory (*t*, *x*_22_(*t*)) of system (66) with the initial values (0.1,0.2,0.5,0.3), (0.6,0.2,0.2,0.9), and (0.3,0.8,0.9,0.7).

Thus, all the conditions in Theorem 11 are satisfied. Then, we can conclude that system (66) has a unique almost periodic solution *x* ^*∗*^(*t*), which is globally exponentially stable. These results are shown in Figures 1 [Fig fig2] [Fig fig3]–4.

## 6. Conclusions

In the article, we have investigated a class of memristor-based shunting inhibitory cellular neural networks with leakage delays. Using the concept of the Filippov solution and differential inclusion, we study the dynamical nature of the memristor-based shunting inhibitory cellular neural networks with leakage delays. Applying a new Lyapunov function technique, a set of sufficient criteria which ensure the existence, uniqueness, and global exponential stability of almost periodic solution of the neural networks are established. The obtained results on the almost periodic solution are applied to prove the existence and stability of periodic solution for this neural network with periodic coefficients and leakage delays. An example is presented to illustrate the effectiveness of the theoretical findings. The almost periodic fluctuations can help us process visual information and predict pathological brain states. At present, pseudo almost periodic solutions of neural networks have also been paid more attention by many authors. However, very few results on pseudo almost periodic solutions of memristor-based neural networks with leakage delays have been reported, which might be our future research topic.
